# Ageing Cattle: The Use of Radiographic Examinations on Cattle Metapodials from Eketorp Ringfort on the Island of Öland in Sweden

**DOI:** 10.1371/journal.pone.0137109

**Published:** 2015-09-03

**Authors:** Ylva Telldahl

**Affiliations:** Osteoarchaeological Research Laboratory, Department of Archaeology and Classical Studies, Stockholm University, Lilla Frescativägen 7, Stockholm, Sweden; Universidade do Algarve, PORTUGAL

## Abstract

In this paper conventional X-ray analysis of cattle metapodials is used to study the age structure of slaughtered cattle at Eketorp ringfort on the island of Öland, Sweden. The X-ray analyses suggest that several animals in both phases were slaughtered aged 4–8 years. More oxen/bulls than cows reached the advanced age of over 8 years, yet in phase III more oxen/bulls seem to have been slaughtered between the ages of 2 and 8 years. These differences may reflect a change in demand for meat related to the character of the site. The results also show a correlation between metapodials with a pathology connected to biomechanical stress and older animals. This suggests that male cattle were used both in meat production and as draught animals. Asymmetry in male metatarsals such as distal broadening of the lateral part of the medial trochlea was visible on the X-ray images. The bone element also indicates a denser outer cortex of the medial diaphysis in comparison to the inner medulla. This could be the result of repetitive mechanical stress. Two metatarsals from cows were documented with distal asymmetry indicating that cows were also used as working animals. Bone elements with changes in the articular surfaces were more common in metapodials from cows with an X-ray age of over 3–4 years. These results highlighted the slaughter age difference between oxen/bulls and cows, enabling a better understanding of animal husbandry and the selection of draught cattle at Eketorp ringfort.

## Introduction

Prehistoric animal husbandry involved the use of cattle for dairying and meat production or as draught animals. The development of ossification in long bones and general growth patterns are commonly used to investigate the slaughter patterns and breeding strategies of the past. Studies by Prummel [1] and Sterba [2] of the identification of foetal skeleton elements have made it possible to separate different species and to identify bones from animals as young as 60 days old. Epiphyseal fusion data are often utilized in the ageing of long bones, together with data on tooth eruption and wear patterns [3–5]. Unfortunately, the morphological analysis of epiphyseal closure is more or less restricted to the identification of four age stages; foetal, unfused, nearly fused and completely fused elements.

X-ray analyses have been used to estimate age and sex and to understand animal exploitation in prehistory [6–14]. In particular the radiographic study by Červený [8]–complemented and described by Kratochvil et al. [10] offers an interesting perspective on the ageing of cattle metapodials. When ossification of the distal trochlea in cattle metapodials is complete, the fusion line disappears, making it only possible to age an element visually as belonging to an animal aged older than 2½ years [4, 5]. The study by Červený [8] shows that changes appear in the growth zone between the diaphysis and epiphysis after the age of 2½ years, making it possible to identify cattle metapodials from animals even older than ten years.

Minerals, protein, size and gross morphology all have different effects on the composition of bone during the life history of an animal [15–18]. Bone consists of inorganic minerals such as calcium and organic material containing lipids, carbohydrates, protein and water [19]. When deposited in soil, the properties of archaeological bone change, as internal and external processes decompose the bone structure [20–24]. A range of diagenetic outcomes can potentially affect the radiographic examination of ancient bones in specific ways. Microbial attack is a major factor, of up to 80%, in collagen loss, compared to almost no histological alteration in temperate regions [25]. Smith et al. [26] studied bone degradation from five different European prehistoric sites with varied contexts (including Sweden), demonstrating a correlation between histological damage and porosity. Furthermore, early taphonomy was found to have a pronounced impact in benign environments and soil conditions had more impact on bone in the long term. Jans et al. studied four different European climatic regimes (including Sweden), finding that fungi might also cooperate with bacterial attack giving signs of “bacterial signature” (21). Favourable environmental factors such as 20% humidity and oxygen increase the risk of fungal attack. Fernandéz-Jalvo et al. demonstrated how factors such as weathering and climate changes diagenetic composition. Their research also found that severe bacterial attack did not affect the external compact bone as much as might be expected and that the collagen was “*generally well preserved*” [20]. Studies of bones in environments with water flow also show that rainwater destroys bone minerals [27, 28]. Karr and Outram [29] documented chemical bone destruction with bone loss in both dry and wet environments. Madgwick and Mulville [30] point out that previous weathering research often used modern bone material. In these cases, the survival biases in comparison with archaeological assemblages are somewhat problematic.

Non-destructive methods such as X-ray images and bone mineral density (BMD) analysis have been used as a tool to understand diagenetic processes and their connection to bone abundance and bone porosity in faunal remains [18, 31–34]. A study of human bone preservation by Gordon and Buikstra [35] showed the importance of external factors; soils with an alkaline pH preserved bones better than acidic soils. Other studies have shown that pH values of 7.8–7.9 preserve bones best [36, 37].

Ageing is an important factor that affects bone structure. If the bone becomes thinner and loses minerals, this weakens the bone matrix and the bone thus becomes more susceptible to fractures. Bone density analysis conducted on bone elements from modern cattle has shown that density values initially increase during maturity but then level off when the metapodials are fused [38]. Yet bone density does not only vary with age [39]. Field et al. [40] investigated metacarpals from Angus or Gelbvieh cows aged 2½-13 years and did not find evidence of increased bone cortex composition with age.

Radiographic studies of modern cattle bones have shown that the calcium content in the skeleton of dairy cows decreases during lactation [41–45]. Mechanical stress induced by compressions or muscles also affect bone density [36, 46]. Chilibeck et al. [47] noted increased bone density when subject to abnormal stress in both humans and animals. Hiney et al. [48] researched immature Holstein bull calves and also demonstrated that high intensity exercise increased metacarpal bone mass. Bartosiewicz et al. [6] used bone density data together with morphological characteristics and X-ray absorption in twenty-five metapodials from modern Romanian draught cattle to show increased distal asymmetry with old age, weight and draught exploitation. Changes in long bone cross-sections due to mechanical stress have also been recorded in donkeys [49].

The distal epiphysial fusion of cattle metapodials from the Eketorp ringfort on the island of Öland in Sweden is analysed here using conventional X-ray images. The purpose of the present study is twofold; firstly, to use radiographic analysis to examine the age structure of the slaughtered cattle and secondly, to discuss the present of pathological conditions in more detail and focus attention on elements that are fully fused. Osteometric measurements on adult cattle metapodials are used to assess size and sex ratio in the slaughtered cattle. The osteometric data have been presented earlier [50–52]. At Eketorp ringfort a number of cattle most probably had been used for draught purposes [53]; thus their metapodials were subject to a mechanical stress.

## Materials and Methods

**Fig 1:**
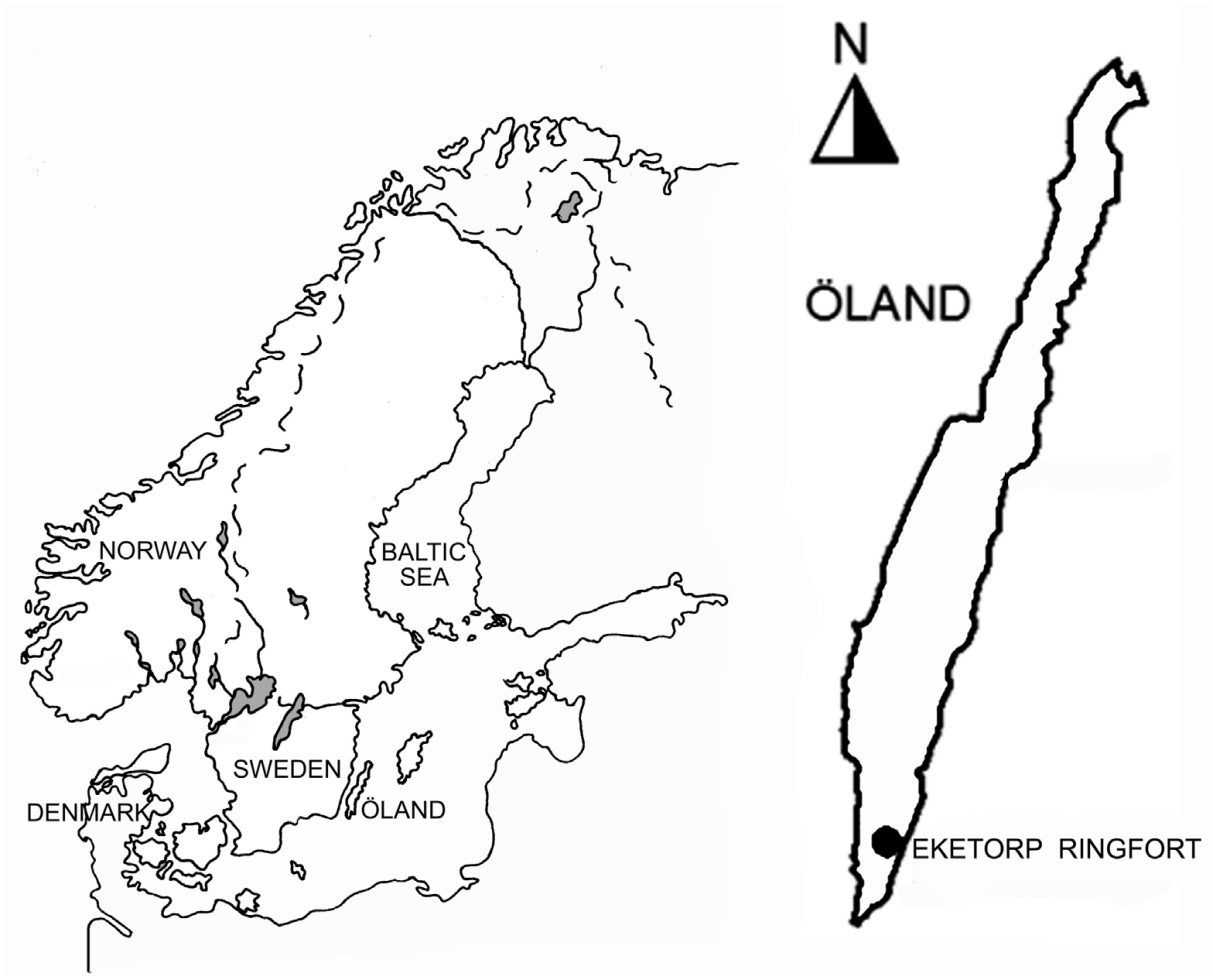
Map showing the location of Eketorp ringfort, Öland island, Sweden.

The animal bones used in this article are food debris from the excavated Eketorp ringfort on the island of Öland in Sweden dated between 300 and 1200/50 A.D. The Museum of National Antiquities, Stockholm, Sweden, has permitted the analysis. Eketorp ringfort is located in the southern part of Öland (Fig 1) and dated from 300-1200/50 A.D. Radiocarbon dating and archaeological observations show three settlement phases: I: 300–400 A.D.; II: 400–650 A.D.; and III: 1170-1200/50 A.D. In the two earliest phases the archaeological finds together with animal bones imply an economy based on husbandry and farming. The third phase represents a more complex ringfort with signs of both military activity and farming settlement. Between phases II and III Eketorp ringfort was abandoned for approximately 450 years [54, 55]. Based on osteometric analysis, the slaughter pattern differs between the phases, showing a slightly higher frequency of older cattle in phase III [52].

Bone preservation at Eketorp ringfort is good and soil analysis has indicated a high alkaline pH [56, 57]. Earlier studies of cattle metapodials from the ringfort show good DNA preservation [51]. Thus it may be expected that internal bone structure would be sufficiently preserved for radiographic analyses to provide accurate results. The metapodials from Eketorp ringfort have previously been studied by Boessneck et al. [52] who identified 2699, both complete and fragmented, cattle metapodials from young and adult animals. A total of 57 metacarpals and 52 metatarsals, complete and fully fused, have been selected for this study (S1 and S2 Tables). The selection criteria focused on fully fused bone elements, present pathology and metapodials that had been genetically sexed and typed [50, 51].

Data on the size and sex ratio of cattle presented in an earlier study was utilized [51] in comparison with radiographic studies by Kratochvil et al. [10] and Červenýs [8] of modern cattle aged 2½-15 years or older. Cattle metapodials develop in three different areas: the third and fourth diaphysis and distal trochlea. The proximal articular facet develops in the foetus while the distal epiphysis is connected to the diaphysis by a growth cartilage that, during maturity, is resorbed [12, 58]. Fusion normally occurs at approximately 2½-3 years.

### Radiographic Photometry

Dorsoplantar projections on metapodials were performed at the Section of Diagnostic Imaging, University Animal Hospital, Swedish University of Agricultural Sciences, Uppsala (Fuji film HR-V, 18x24 cm, Screen 100, exposure KV 52, MAS 5.1, with 90 cm focal distance). VetTech GeR radiology equipment was used. Interpreting a plane X-ray image is complex since it mirrors many different angles of the bone element. For this reason, the X-ray projection of a growth plate might show a “thicker” white contour than the actual “thickness”. In dry metapodia, depending on age at slaughter, the structure from the distal growth plate leaves a different imprint visible on the X-ray images. The classification of X-ray images follows the description by Červenýs [8] and Kratochvil et al. [10] with radiographic images in six age groups (AG) as shown in Table 1.

**Table 1:** Morphological changes and their classification of age categories into radiographic images. Description and quotations from Ĉervený [8] and Kratochvil et al. [10]. Age Group 1 (0–2 years) is excluded in the present study since only fully fused metapodials were examined.

Age group(AG)	Age(years)	Description and quotations
1	0–2	Light line revealing a disappearing growth cartilage. In this stage the diaphysis andepiphysis of metapodials are separated when found in archaeological assemblages. Therefore this AG is excluded in this study.
2	2–3	(a-b) The bright line following the epiphyseal-diaphyseal cartilage is visiblemerely along the perimeter of the epiphyseal-diaphyseal zone, outer growth zonesare protruding from the bone surface (c) (Fig 2).
3	3–4	Shaded symphysis that is protruding from the bone surface (Fig 3).
4	4–8	Narrow symphysis that is indistinct from the bone surface, spongiosa (Fig 4).
5	8–14	Shaded symphysis that is level with the bone surface (Fig 5).
6	>15	Reduced line of symphysis and the distal part of the metapodium has a conicalshape (Fig 6).

**Fig 2:**
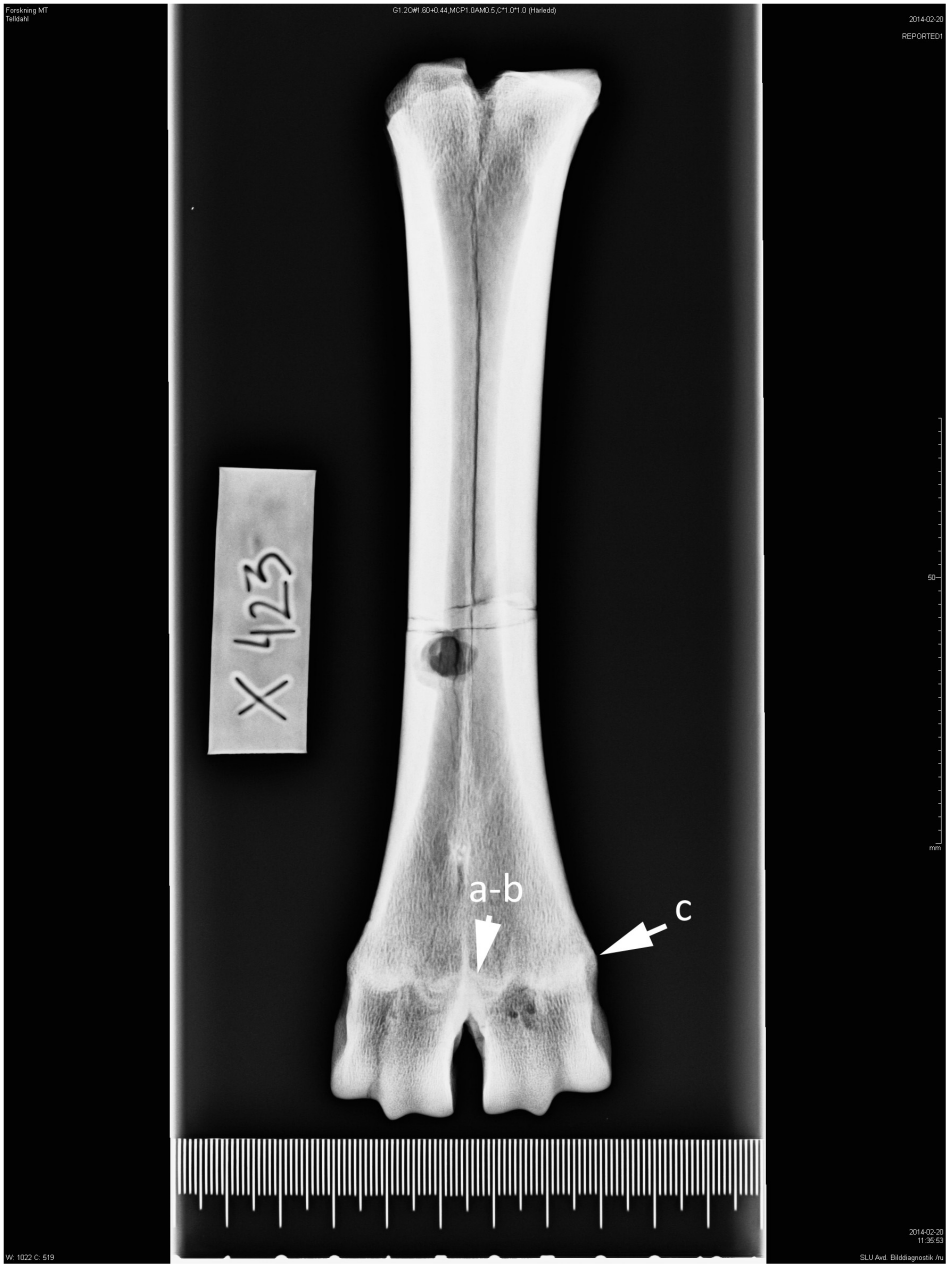
Right-sided metatarsal in dorsoplantar projection. Ox/bull. Age group II—cattle 2–3 years. (a) The irregular bright line following location of the epiphyseal plate of cartilage, (b) epiphyseal and (c) diaphyseal bone plate, (d) outer circumference of epiphysial-diaphysial growth zones are protruding (showing a convex outer margin) from the bone surface.

**Fig 3:**
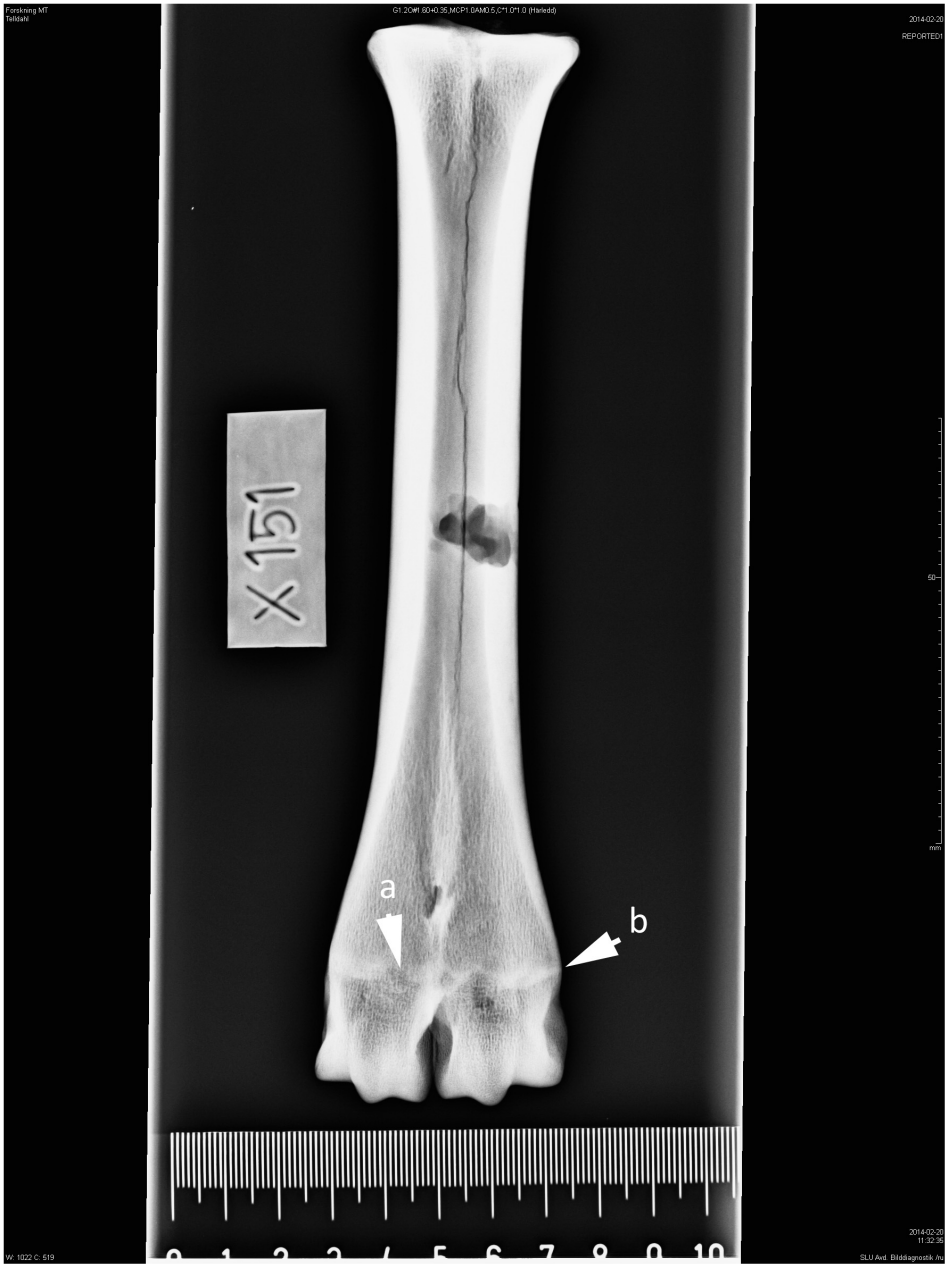
Left-sided metatarsal in dorsoplantar projection. Cow. Age group III—cattle 3–4 years. (a) Shaded symphysis, which is (b) protruding from the bone surface.

**Fig 4:**
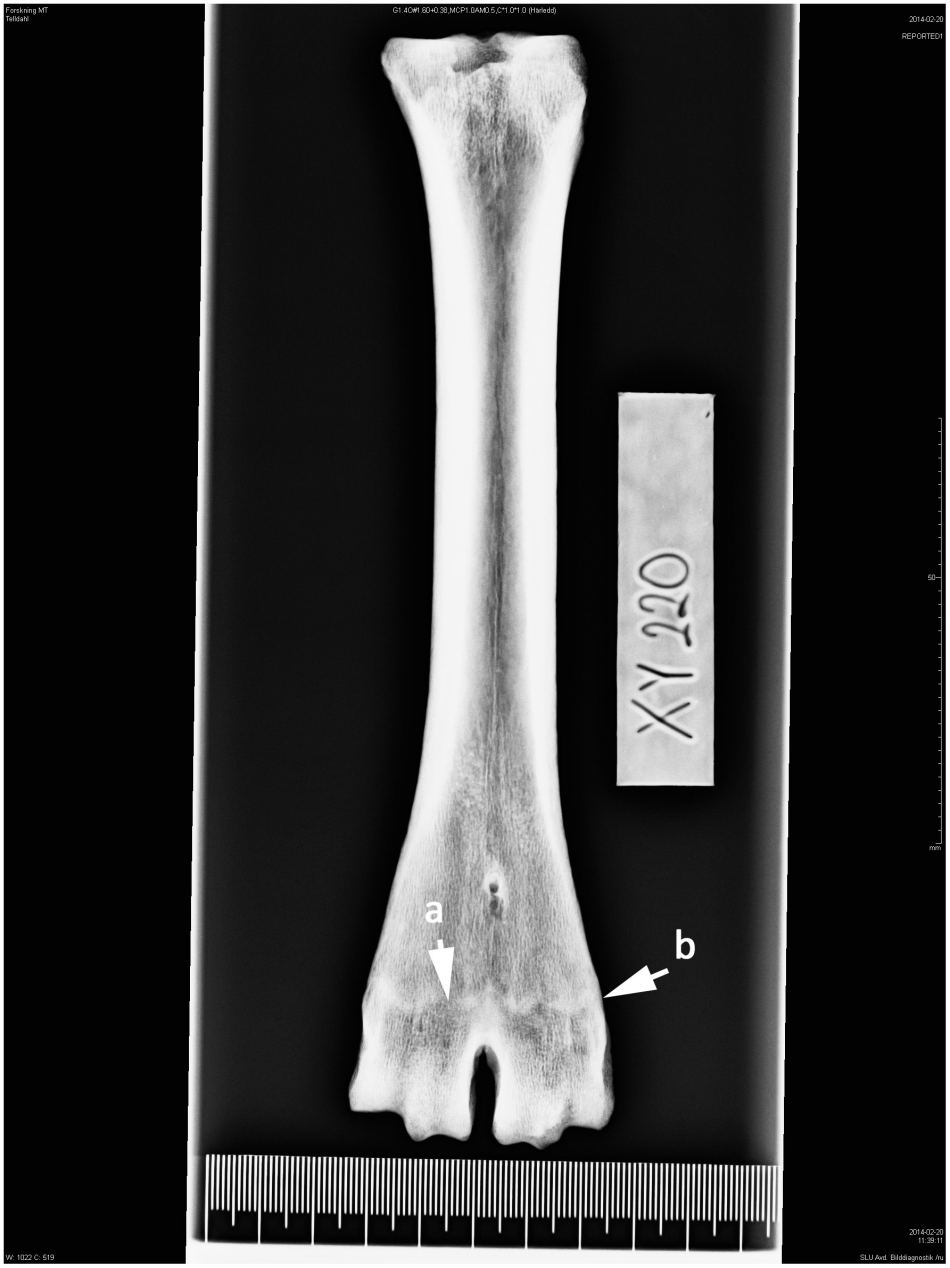
Right-sided metatarsal in dorsoplantar projection. Cow. Age group IV—cattle 4–8 years. (a) Narrow symphysis that is (b) indistinct from the bone surface.

**Fig 5:**
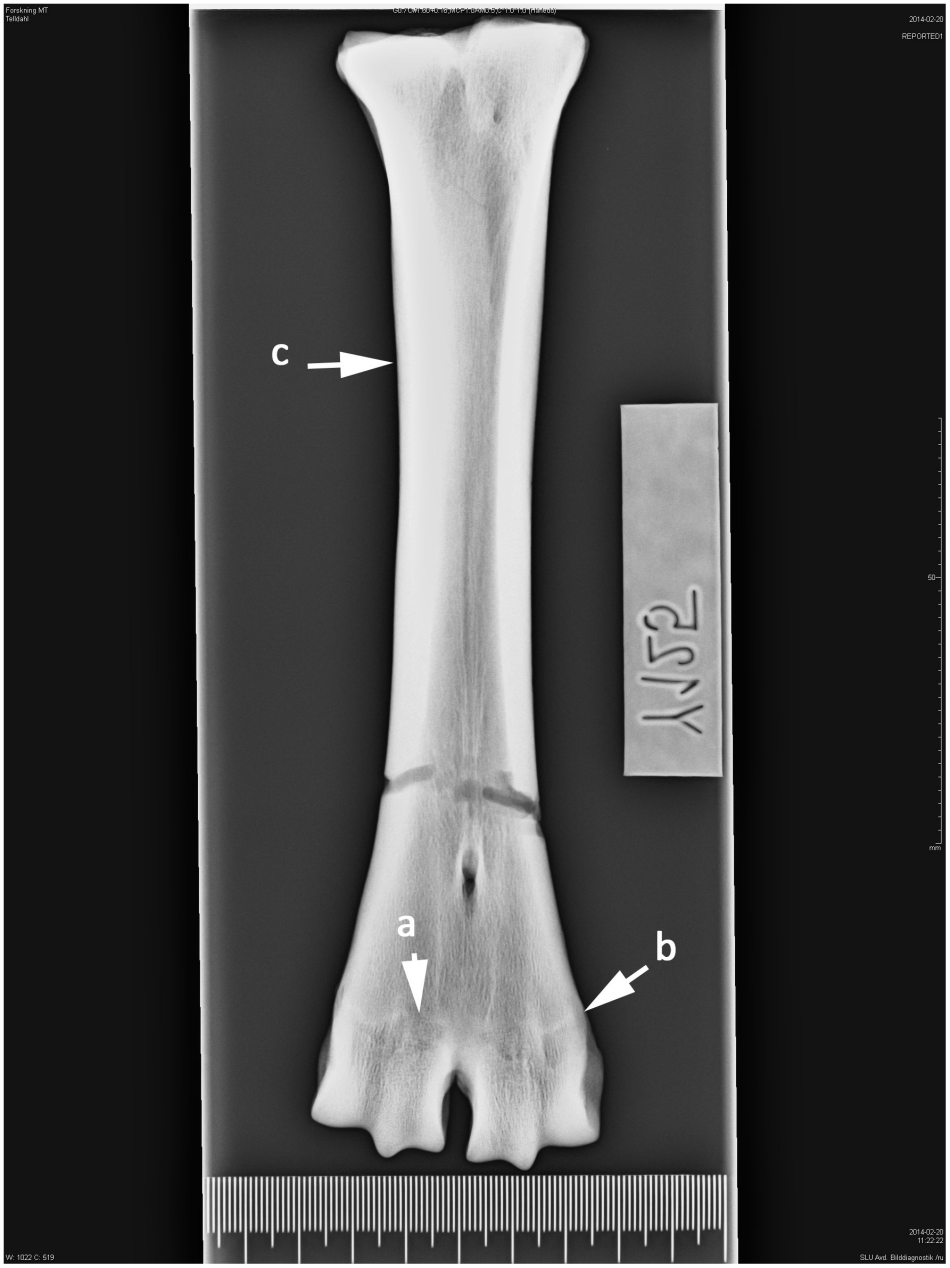
Right-sided metatarsal in dorsoplantar projection. Ox/bull. Age group V—cattle 8–14 years. (a) Thin shadow of the epiphysial-diaphysial symphysis that is (b) level with the bone surface, (c) thickening of the diaphysis probably due to bone inflammation, see [53].

**Fig 6:**
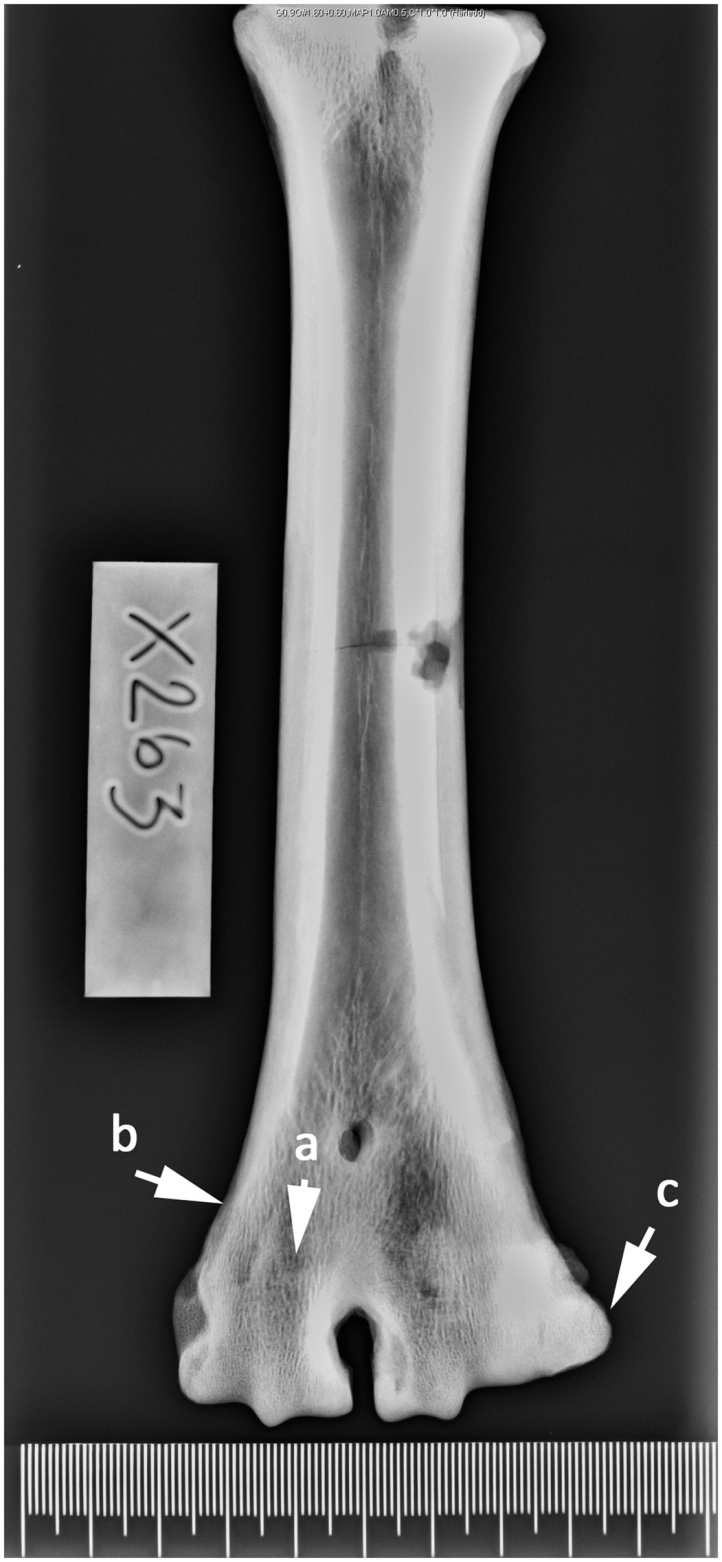
Right-sided metatarsal in dorsoplantar projection. Ox/bull. Age group VI—cattle 15 years and over. (a) Reduced line of symphysis and (b) the distal part of metapodium have a conical shape, (c) broadening of the medial trochlea and exostosis on distal plantar bone surface [53].

These studies show that in seven-month-old calves, secondary ossification centres have developed in the long bones (growth plates). These centres consist of osteoblasts responsible for bone length. Growth plates are relatively easy to recognize on an X-ray image because they are softer and contain less mineral than the surrounding bone, making them appear darker. As the animal grows, the epiphyseal-diaphyseal cartilages change in appearance on the X-ray images. At a young age, they exhibit irregular patterns with high topography. After distal epiphyseal fusion these patterns leave traces in the dry bone, seen as shaded white contours on the X-ray images (Fig 2). From the second year the growth cartilages become narrower and “*epiphyseal bony plates of growth zone fused after destruction of growth cartilage to form concentration of osseous trabeculae of epiphyseal-diaphyseal scar*”. When the animals have reached the age of 6 years or older the “*the demarcation line of the epiphyseal-diaphyseal scar is obscure due to rebuilding that had occurred in the bones*”. When the animals have reached 15 years of age the osseous trabeculae of epiphyseal-diaphyseal scar has resorbed and its contour is no longer visible in the dry bone [8]. The distal parts of the diaphysis also changes shape during skeletal maturity, which is visible both to the naked eye and on the X-ray images. At a young age the outer margin of the symphysis protrudes from the bone surface showing a convex shape (Fig 3). Kratochvil et al. [10] recorded that between the ages of 8–14 years the outer margin become more level with the bone surface and after 15 years of age the distal end of the metapodium became conical in shape (Fig 6).

## Results

On the basis of the growth stage of the epiphysis and diaphysis, 64% of the metapodials come from animals aged 2–8 years (AG 2–4). Overall, the age structure is similar in the front and hind limbs; 60% of the metacarpals and 66% of the metatarsals belong to AG 2–4 (Table 2, S1 and S2 Tables).

**Table 2:** Age structure based on X-ray examination. Stage 1 (AG 0–2 years) is excluded since only fully fused metapodials were examined in the present study. l/r = left/right bone element.

	N	Age groups (AG)				
	l/r	2	3	4	5	6
Phase II						
Metacarpals	5/6	0/1	0/1	1/1	3/2	1/1
Metatarsals	3/6	1/1	1/0	0/1	1/4	0/0
Phase II/III						
Metacarpals	4/7	0/2	1/3	1/0	2/2	0/0
Metatarsals	4/4	1/0	0/1	2/2	1/1	0/0
Phase III						
Metacarpals	17/18	0/2	4/2	7/8	3/5	3/1
Metatarsals	13/22	1/1	5/4	4/8	1/5	2/4

Shaded irregular lines (high topography, zig-zag pattern) at the centre of the growth zones with a brighter line at the outer part of the zones were recognized on ten metapodials and correspond to newly fused bone elements without evidence of an ocular fusion line (AG 2). The radiographic images of two newly fused metacarpals showed that the growth cartilage between the diaphyseal and epiphyseal bone plates had disappeared recently, but ocular fusion lines were evident.

Absences of growth cartilage plate together with a dense shadow indicate that bone growth is complete. The outer margins of the symphysis were still protruding from the bone surface (Fig 3). This was observed on 20.2% of the metapodials and corresponds to an age of between 3 and 4 years (AG 3). 34.5% of the bone elements have a symphysis with more distinction at the lateral and medial border but with a narrower epiphyseal-diaphyseal symphysis, showing animals that have reached AG 4 (Fig 4). Approximately 27% of the metapodials were classified as AG 5 (Fig 5). In those bones the shadow of the epiphyseal-diaphyseal symphysis was visible but its outer part was indistinct and no longer protruding from the bone surface. Twelve metapodials showed signs of resorption of the epiphyseal-diaphyseal symphysis identified as thinner protruding parts, indicating animals in AG 6. In this age group the outer part of the diaphysis had become more uniform with the bone surface and the distal trochlea showed the more conical shape recognized in adult animals.

There is an interesting difference in the age distribution between phases II and III (Table 2). In phase II there is an indication that cattle reached older age groups before they were slaughtered. In phase III the majority of slaughtered cattle belonged to younger age groups. 19 metapodials belong to the uncertain phase II/III and their age distribution resembles phase III, with thirteen recorded in AG 2–4, of which 10 belonged to cattle aged 3–8 years, and six to AG 5.

### Pathology, Age and Sex

This research indicates a correlation between type of pathology, X-ray age and sex (S1 and S2 Tables). Distal asymmetry was recorded in 21.8% of the metapodials, of which 14.6% belong to oxen/bulls over 8 years of age. Only two metatarsals with distal asymmetry belong to cows aged between 3–4 and 8–14 years. The majority of skeletal lesions in cows is depressions in the articular facets [50], recorded in 17.1% of metapodials over 8 years but also in the younger 3–4 age group. One cow metatarsal from an animal in the highest X-ray age group had several skeletal changes such as proximal exostosis at the dorsal aspect of the medial articular facet including two depressions, one in the dorsoplantar direction (13.5 mm long and 2.3 mm broad) and one needlepoint depression on the medial articular facet. Three metacarpals from cows had ossification of the interosseous ligaments between the metapodial shaft of IV and V, with X-ray ages between 4–8 and 8–14 years. No correlation was recorded between bone elements without pathology and age: 35.5% belonged to animals over 4 years of age.

## Discussion

Radiographic images may be valuable tools for assessing the age structure of slaughtered animals in archaeological assemblages. X-ray analysis complements the interpretations of cattle utilization patterns at Eketorp ringfort. The results indicate a difference between the phases in that the age structure in phase II corresponds more to a farming settlement whereas phase III, with its higher frequency of slaughtered cows and oxen/bulls aged 3–8 years, corresponds to a greater demand for meat. This is probably associated with the different character of the site in the two phases. The inhabitants in phase III were probably more dependent on imported meat and it seems that the surrounding farms to some extent specialized in providing the ringfort with resources, or were forced to provision it.

**Fig 7:**
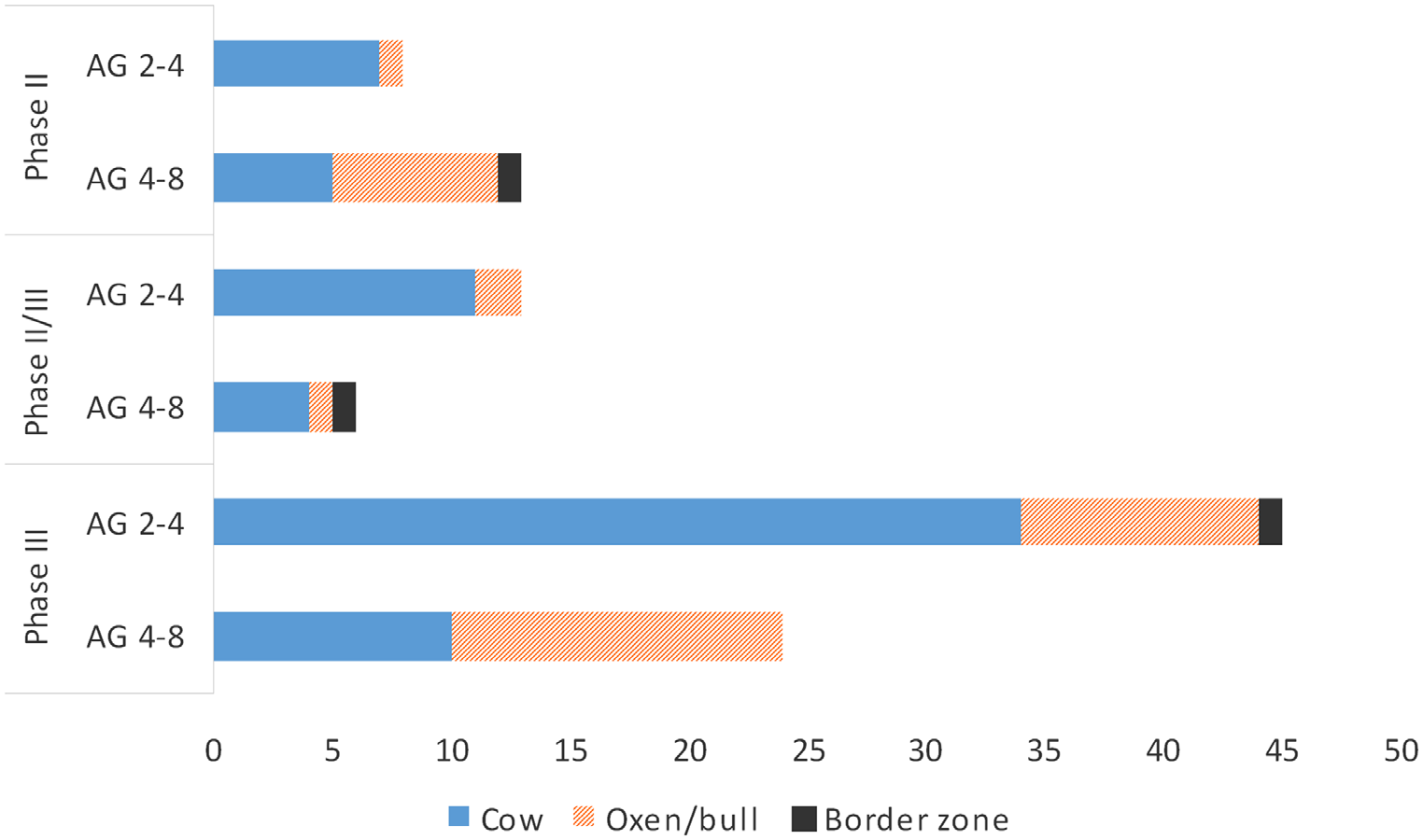
Age group in relation to sex on the basis of distal breadth of the metapodials from Eketorp ringfort, Öland Island, Sweden.

The sex ratio of the metapodials in relation to age is compared using the distal breadth measurement (Bd) of 57 metacarpals and 52 metatarsals (Fig 7, Table 3).

**Table 3:** Age groups in relation to sex based on the distal breadth of the metapodials. The *border zone* (no sex) between cows and oxen/bulls is from 53.5 to 54.5 mm for the metacarpals and between 50.5 and 52 mm for the metatarsals, according to Telldahl et al. (2012).

	*n*	Phase II		Phase II/III		Phase III	
		AG 2–4	AG 5–6	AG 2–4	AG 5–6	AG 2–4	AG 5–6
Metacarpals							
Cow	42	4	4*	7	3	18*	6
Border zone	*2*				*1*	*1*	
Ox/bull	13		3			4	6
**Total**	**57**	**4**	**7**	**7**	**4**	**23**	**12**
Metatarsals							
Cow	29	3	1	4	1	16	4
*Border zone*	*1*					*1*	
Ox/bull	22	1	4	2	1	6*	8
**Total**	**52**	**4**	**5**	**6**	**2**	**23**	**12**

* Sex estimation on three metacarpals (S1 Table: Id 226, 342 and 414) and one metatarsal (S2 Table; Id 214) is based on previous biomolecular analysis (see S1 and S2 Tables and Telldahl et al., 2012).

**Fig 8:**
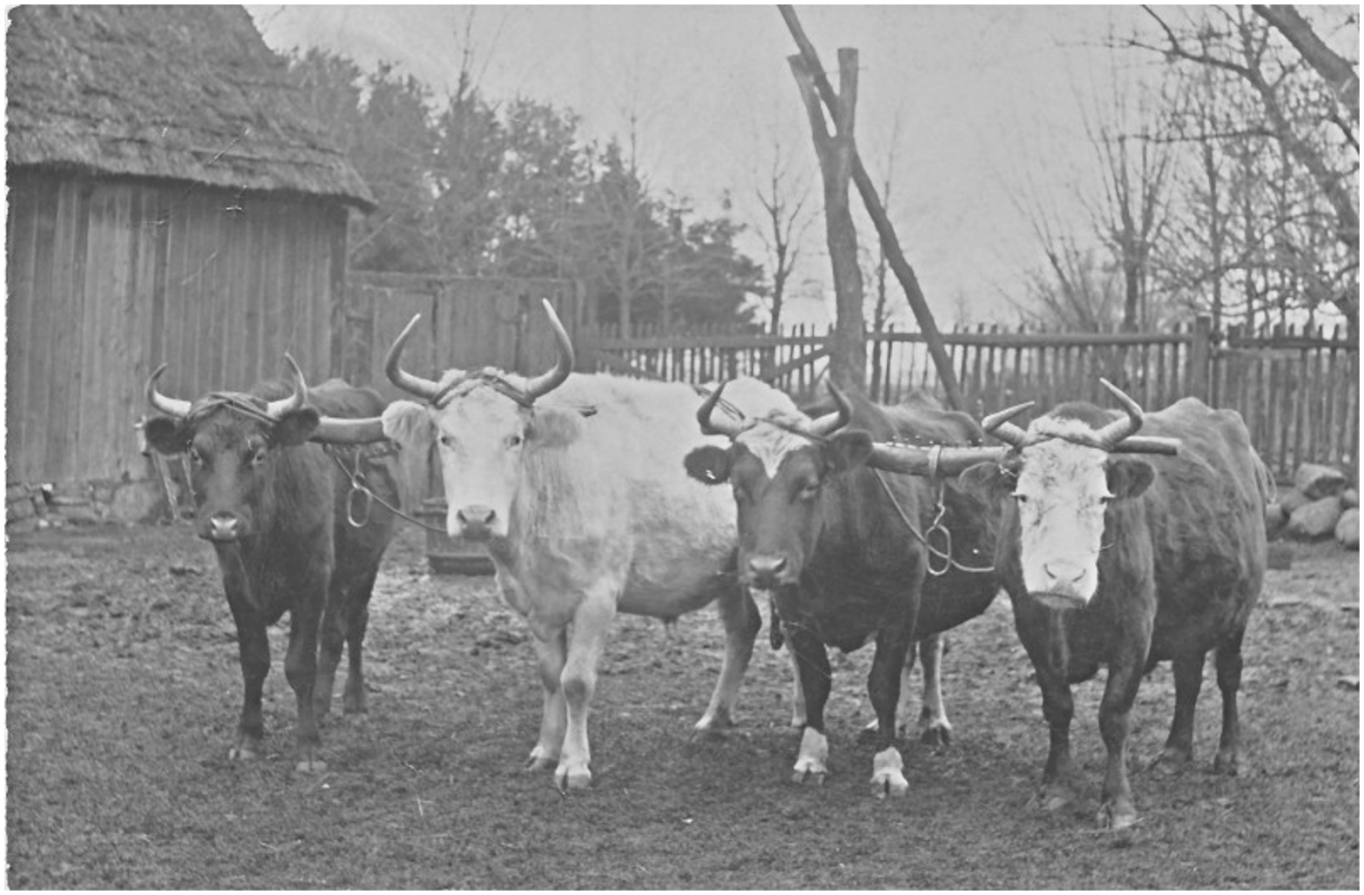
Draught oxen from Skedstad Parish on Öland Island in Sweden, at the beginning of the 19^th^ century. Reproduced with permission of Astrid Westerman, Bredsättra Local History Society, Öland, Sweden.

There are interesting similarities as well as differences in the slaughter pattern for cows and oxen/bulls between phases II and III. In general, few cows reached an advanced age (AG 5–6) since many animals seem to have been slaughtered before the age of 4–8 years in both phases. More oxen/bulls (older than 2–3 years) reached an advanced age (AG 5–6). The kill pattern with high percentage of young calves in phase III at Eketorp ringfort is thought to indicate meat production for the ringfort [52]. The metric data on 1150 completely fused bone elements showed that approximately 75% of the cattle were cows and only two oxen were identified [52](S3 Table). The latter two were identified by the longest metatarsals, one of which had distal broadening of the lateral part of the medial trochlea. The X-ray images suggest that this bone element belonged to an animal between 4 and 8 years of age. Prehistoric breeding and change in body size have been proposed to indicate improved livestock [59]. At Eketorp ringfort the cattle in phase III was of approximately 2 cm greater withers height than in phase II [52]. The ringfort was abandoned for approximately 450 years between the two phases and it has been debated whether a new breed was introduced in phase III. Yet the previous study of genetic characteristics and cattle body size suggests gradual specialization in meat production in phase III and that the cattle population was of a homogeneous breed [50]. This probably mirrors a greater demand for meat in phase III [54, 60]. In the modern specialized breeding system cows are most productive between the ages of 4 and 9 years (in crossbreeds, between 5 and 10 years) during which a calcium supplement is given to cows in order to avoid the bone mineral loss known as parturient paresis (milk fever). This is based on pregnancy rates when cows are used for breeding from 2 years of age and produce approximately one calf per year until they are worn out and thereafter only good for meat [61, 62]. Cows from the medieval town of Skara are believed to have produced a total of 3–4 calves each [63]. Swedish historical sources such as tax rolls and records from the estate accounts of 16^th^ century royal manors indicate that not all cows produced calves annually and not all calves survived. This could probably be due to low quantities of fodder. Calving and milking was distributed throughout the year and during winter the cows were stalled [64]. The tax rolls also recorded that during the late medieval period, ox drives from the southern part of Sweden, including Öland, were well established. More than a hundred oxen were transported from Öland in the years 1572–73 [65]. At the beginning of the 19^th^ century draught oxen were still the most common animal used in Ölandic farming (Fig 8).

The relationship between metapodial morphology, growth and nutrition in sheep has been demonstrated by Tschirvinsky [66] in the early 20^th^ century, reiterated mid-century by Hammond, and examined in more detail by Pálsson and Verges [67]. Popkin et al. [68] conducted large scale research on unimproved Shetland sheep and also recorded a correlation between low nutrition and more prolonged growth period in comparison with animals with high nutritional intake that led to fusion earlier in life. Kratochvil et al. [10] considered the possibility that fusion of the metapodia in medieval cattle might occur 1–2 years later than in modern breeds. Another relevant issue is whether castration may affect the appearance of the metaphyseal area. A longer fusion period of 2–3 years due to castration in medieval oxen was therefore accepted by Kratochvil et al. A recent histological study has shown that the growth layer at the periosteum is thicker in castrates than in cows and bulls [69]. Early castration delays the distal epiphyseal fusion of metapodials and consequently oxen became larger than bulls and cows [70]. Castrated bulls may exhibit X-ray growth plate images characteristic of a younger age. This has to be considered to some extent in explaining the difference in the slaughter patterns of oxen/bulls between phases II and III. If castration was more common in phase III this may affect the patterns observed. In this study two large metatarsals belonged to animals of approximately 15 years of age. Our previous molecular study showed that the elements come from males and thus probably from castrates [51]. Historical sources from the Roman period often recommend castration of male cattle when the animal has reached ontogenetic age [71]. Variations in maturity cannot be ruled out for individual cattle at Eketorp ringfort. A recent study by Johannsen [72] showed that the exploitation of cattle often follows the farm size, nature of the tasks and ideological preferences that can vary within one cultural context. During phase II the Eketorp ringfort economy was based on husbandry and farming, in contrast to the fortified complex in phase III. During phase III the majority or at least part of the meat was probably delivered to the ringfort from the surrounding farms [52, 55, 60]. The molecular data on genetic variation in cattle breeding showed that all but two male cattle belonged to haplogroup Y2 both within each phase and between phases II and III [50]. Thus the general maturity of the Eketorp cattle was probably homogeneous.

The possibility of biological attacks and their impact on the distal epiphyseal fusion area in metapodials have been taken into consideration. According to Jans et al. [21] fungal and bacterial attack alter both organic content and histological microstructure. Four types of tunnelling are described as destroying the internal bone matrix. These empty tunnels with well-defined calcified walls are 1.2–15 μm in diameter and not visible on conventional X-ray images. However, it is 'highly unlikely' that the tunnelling would show any correlation with the biomechanical organisation of the bone and, therefore, unlikely to resemble spongiosa (Personal communication Dr Sonia O´Connor).

With age the epiphyseal-diaphyseal cartilages change in appearance and are replaced by osseous tissue between the diaphyseal and epiphyseal bone plates. On the X-ray images the osseous scar is seen as irregular patterns, with the high topography seen in the young animal becoming narrower with age and eventually resorbed [10]. Bones are better preserved in calcareous soil than in an acidic environment. Calcareous soil conditions can be found on the islands Gotland and Öland, whereas the soil on the west coast of the Swedish mainland contains more acidifying pollutants [57, 73]. The soil environment and previous genetic results at Eketorp ringfort indicate good preservation [50, 51].

According to Columella [71] the distal extremities, shoulders and horns were all subject to injuries when Roman cattle were used as draught animals. Although the front limbs carry 60% of an animal’s weight [74], the majority of skeletal lesions is recorded in the hind limb [53]. Bartosiewicz et al. [70] found a correlation between age, traction and distal asymmetry in modern draught oxen. The majority of metapodials with distal asymmetry were recorded in bone elements with an X-ray age of over 4–8 years, suggesting that both oxen/bulls and a few cows were used for traction. The use of cows in traction work impacts negatively on both meat and milk production. The total percentage of skeletal lesions in bone elements from the extremities showed a correlation to large sized or robust animals. Yet not all large bone elements were recorded with lesions [53]. Bendrey [75] documented a correlation between old age in horses and ossification of the interosseous ligaments between the metapodial shafts. This might also be the case in four cow metacarpals from Eketorp with X-ray ages over 4–8 years. Furthermore, ossification of the second metacarpal was never recorded in young bulls by Bartosiewicz et al. [76]. The biomechanics of the mesaxonic equid foot and the paraxonic cattle foot are sufficiently different, however, and the parallels have not been fully investigated.

Age is a factor that could increase the development of skeletal lesions. Yet in this study 59.9% of the metapodials were not pathological and 72.2% of these had an X-ray age of over 4–8 years. Changes in the articular surfaces were in the majority in metapodials from cows, some of which were indicative of spavin [53]. Bartosiewicz et al. [70] discuss “narrow hocks” and “cow hocks” as inherited properties that eventually could weaken the hock joint in draught animals. If the Eketorp cows belonged to a local breeding population, this increases the risk of a hereditary weak skeletal constitution. In contrast, factors such as the ontogenetic age at which animals are selected and the duration of cattle work life will also affect how the metapodials withstand biomechanical stress.

## Conclusions

**Fig 9:**
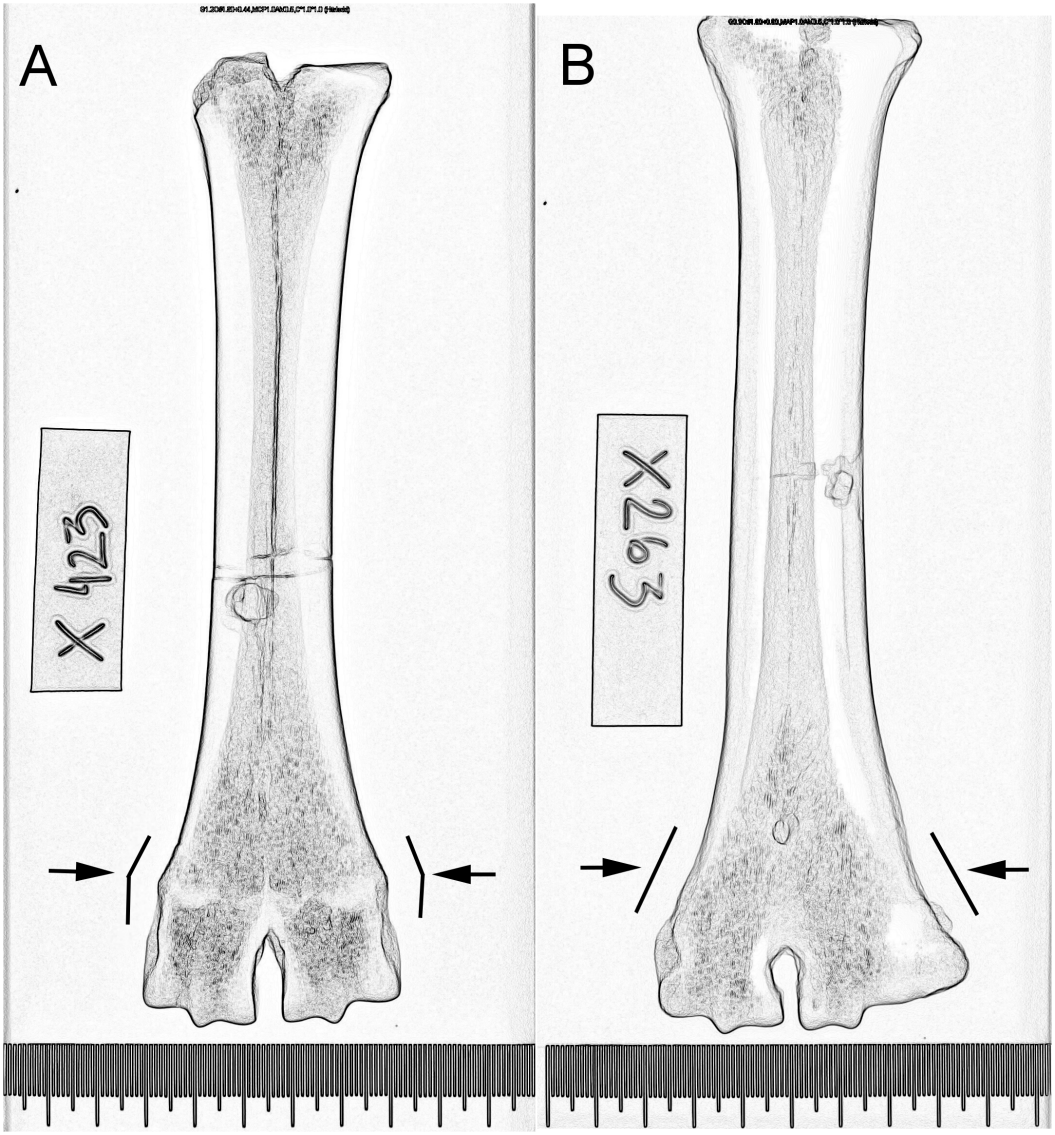
Stylized contourfilter of X-ray images (Figs 3 and 6) of cattle metatarsals from Eketorp ringfort. A: Os metatarsal in Age Group 3 with distal convex outer margin (arrows). B: Os metatarsal in Age Group 6 with conical outer margin (arrows).

Conventional X-ray analysis of cattle metapodials was used to study the age structure of slaughtered cattle at Eketorp ringfort on the island of Öland, Sweden. The X-ray analyses show that few cows reached an advanced age (AG 5–6) while many animals seem to have been slaughtered aged 4–8 years in the last two settlement phases. More male cattle (older than 2–3 years) reached over 8 years old but in phase III more male cattle seem to have been slaughtered aged between 2 and 8 years. Changing castration patterns may, however, affect the interpretation. The differences probably reflect a shift in demand for meat related to the changing character of the site. The analysis has also shown that the distal shape of metapodials is helpful when X-ray is not possible. The outer perimeter of the epiphyseal-diaphyseal symphysis in young animals is more convex (prodtruding) and becomes more conical with age (Fig 9A and 9B). Further research could combine X-ray analysis with both molecular and chemical analysis on a larger series of similar data to study the transition towards specialization and trade in animals in prehistory.

## Supporting Information

S1 Table Descriptive X-ray data on 57 metacarpals. Age groups are presented in section Material and methods and Table 1. Bone Id = Id number at the Museum of National Antiquities, Stockholm, Sweden. l/r = left/right bone element. GL = greatest length, Bd = distal breadth. Pat y/n = the presence of pathology yes/no. Sorting by Age Group and phase.
(DOCX)

S2 Table Descriptive X-ray data on 52 metatarsals. Age groups are presented in section Material and methods and Table 1. Bone Id = Id number at the Museum of National Antiquities, Stockholm, Sweden. l/r = left/right bone element. GL = greatest length, Bd = distal breadth. Pat y/n = the presence of pathology yes/no. Sorting by age range and phase.
(DOCX)

S3 Table Summarized data based on Boessneck et al (1979:66, diagram 7) measurements on bone elements from Eketorp ringfort phase II and III. (DOCX)
