# Supplementary material for: Ageing Cattle: The Use of Radiographic Examinations on Cattle Metapodials from Eketorp Ringfort on the Island of Öland in Sweden
Source: PLoS One. 2015 Sep 3;10(9):e0137109. doi: 10.1371/journal.pone.0137109 (PMC4559407; doi:10.1371/journal.pone.0137109)
Supplement: S1 Table — Age groups are presented in section Material and methods and Table 1. Bone Id = Id number at the Museum of National Antiquities, Stockholm, Sweden. l/r = left/right bone element. GL = greatest length, Bd = distal breadth. Pat y/n = the presence of pathology yes/no. Sorting by Age Group and phase. (DOCX) [file pone.0137109.s001.docx]

S1 Table: Descriptive X-ray data on 57 metacarpals. Age groups are presented in section

Material and methods and table 1. Bone Id= Id number at the Museum of National

Antiquities, Stockholm, Sweden. l/r= left/right bone element. GL= greatest length,

Bd= distal breadth. Pat y/n= the presence of pathology yes/no. Sorting by Age Group

and phase.

| **Phase** | **Bone**  **Id** | **l/r** | **Pat**  **y/n** | **X-ray**  **age** | **Age**  **group** | **GL** | **Bd** |
| --- | --- | --- | --- | --- | --- | --- | --- |
| II | 274 | r | y | 2-3 | 2 |  | 52,79 |
| II | 168 | r | n | 3-4 | 3 | 170,71 | 47,44 |
| II | 124 | r | y | 4-8 | 4 | 181,76 | 51,54 |
| II | 1n | l | n | 4-8 | 4 | 192,09 | 53,38 |
| II | 1h | l | y | 8-14 | 5 | 187,81 | 51,14 |
| II | 414 | l | n | 8-14 | 5 | 181,85 |  |
| II | 342 | r | n | 8-14 | 5 | 186,3 | 53,69 |
| II | 368 | r | y | 8-14 | 5 |  | 58,99 |
| II | 143 | l | y | 8-14 | 5 |  | 56,79 |
| II | 150 | r | n | 15 | 6 | 186,53 | 51,63 |
| II | 193 | l | n | 15 | 6 | 177,07 | 56,09 |
| II/III | 168 | r | y | 2-3 | 2 |  | 48,93 |
| II/III | 294 | r | n | 2-3 | 2 | 175,89 | 48,5 |
| II/III | 101 | r | n | 3-4 | 3 | 181,39 | 45,79 |
| II/III | 319 | r | n | 3-4 | 3 | 188,47 | 50,76 |
| II/III | 317-318 | r | n | 3-4 | 3 | 186,09 | 49,74 |
| II/III | 79 | - | y | 3-4 | 3 |  | 50,17 |
| II/III | 324 | l | n | 4-8 | 4 | 177,08 | 48,89 |
| II/III | 101 | r | n | 8-14 | 5 | 171,77 | 49,54 |
| II/III | 145 | r | n | 8-14 | 5 | 186,09 | 51,14 |
| II/III | 167 | l | y | 8-14 | 5 | 173,13 | 48,37 |
| II/III | 268 | l | n | 8-14 | 5 | 184,09 | 53,59 |
| III | 189 | r | n | 2,5* | 2 | 176,09 | 54,23 |
| III | 194 | r | y | 2-3 | 2 |  | 49,27 |
| III | 141 | r | y | 3-4 | 3 | 177,49 | 50,3 |
| III | 174 | l | n | 3-4 | 3 | 186,93 | 52,65 |
| III | 174 | r | n | 3-4 | 3 | 190,2 | 52,32 |
| III | 398 | l | n | 3-4 | 3 | 175,67 | 55,88 |
| III | 192 | l | y | 3-4 | 3 | 181,2 | 58,77 |
| III | 174 | l | y | 3-4 | 3 |  | 60,13 |
| III | 201 | r | n | 4-8 | 4 | 180,02 | 47,33 |
| III | 394 | r | y | 4-8 | 4 | 177,76 | 48,07 |
| III | 169 | r | n | 4-8 | 4 | 176,49 | 49,36 |
| III | 349 | l | y | 4-8 | 4 |  | 50,36 |
| III | 169b | r | n | 4-8 | 4 | 181,7 | 47,78 |
| III | 291 | r | n | 4-8 | 4 | 187,66 | 50,58 |
| III | 120 | l | y | 4-8 | 4 |  | 51,3 |
| III | 99 | l | n | 4-8 | 4 | 182,3 | 51,11 |
| III | 145 | r | n | 4-8 | 4 | 189,77 | 51,34 |
| III | 173 | r | n | 4-8 | 4 | 189,01 | 51,44 |
| III | 373 | l | n | 4-8 | 4 | 185,05 | 52,45 |
| III | 412 | l | n | 4-8 | 4 | 187,87 | 53,12 |
| III | 174 | r | n | 4-8 | 4 | 166,36 | 52,7 |
| III | 367 | l | y | 4-8 | 4 |  | 60,46 |
| III | 226 | l | y | 4-8 | 4 |  |  |
| III | 293 | l | n | 8-14 | 5 | 180,82 | 51,11 |
| III | 252 | r | n | 8-14 | 5 | 180,92 | 50,74 |
| III | 14 | r | y | 8-14 | 5 | 177,56 | 50,34 |
| III | 165 | r | y | 8-14 | 5 | 182,49 | 59,91 |
| III | 252 | l | y | 8-14 | 5 |  | 61,39 |
| III | 214 | l | y | 8-14 | 5 | 188,75 | 64,76 |
| III | 165 | r | y | 8-14 | 5 | 189,79 | 62,61 |
| III | 362 | r | y | 8-14 | 5 | 183,76 | 49,57 |
| III | 1g | r | n | 15 | 6 | 184,27 | 50,06 |
| III | 172 | l | n | 15 | 6 | 197,49 | 52,58 |
| III | 168 | l | n | 15 | 6 | 185,3 | 59,69 |
| III | 321 | l | y | 15 | 6 | 170,59 | 59,2 |

*Visible line of fusion
