# Supplementary material for: Ageing Cattle: The Use of Radiographic Examinations on Cattle Metapodials from Eketorp Ringfort on the Island of Öland in Sweden
Source: PLoS One. 2015 Sep 3;10(9):e0137109. doi: 10.1371/journal.pone.0137109 (PMC4559407; doi:10.1371/journal.pone.0137109)
Supplement: S2 Table — Age groups are presented in section Material and methods and Table 1. Bone Id = Id number at the Museum of National Antiquities, Stockholm, Sweden. l/r = left/right bone element. GL = greatest length, Bd = distal breadth. Pat y/n = the presence of pathology yes/no. Sorting by age range and phase. (DOCX) [file pone.0137109.s002.docx]

S2 Table: Descriptive X-ray data on 52 metatarsals. Age groups are presented in section

Material and methods and table 1. Bone Id= Id number at the Museum of National

Antiquities, Stockholm, Sweden. l/r= left/right bone element. GL= greatest length,

Bd= distal breadth. Pat y/n= the presence of pathology yes/no. Sorting by age range

and phase.

| **Phase** | **Bone Id** | **l/r** | **Pat**  **y/n** | **X-Ray age** | **Age**  **group** | **GL** | **Bd** |
| --- | --- | --- | --- | --- | --- | --- | --- |
| II | 148 | r | y | 2,5* | 2 | 199,03 | 47,25 |
| II | 327 | l | y | 2-3 | 2 | 206,96 | 57,03 |
| II | 150 | l | n | 3-4 | 3 | 199,12 | 43,72 |
| II | 147 | r | n | 4-8 | 4 | 202,66 | 46,12 |
| II | 125 | r | y | 8-14 | 5 | 213,51 | 53,56 |
| II | 168 | r | n | 8-14 | 5 | 203,47 | 43,31 |
| II | 199 | r | y | 8-14 | 5 |  | 59,46 |
| II | 245 | l | y | 8-14 | 5 | 196,5 | 57,85 |
| II | 465 | r | y | 8-14 | 5 |  | 57,38 |
| III/II | 220 | l | n | 2-3 | 2 | 204,44 | 49,6 |
| III/II | 119 | r | y | 3-4 | 3 | 210,17 | 53,03 |
| III/II | 224 | l | y | 4-8 | 4 | 228,22 | 55,25 |
| III/II | 297 | r | n | 4-8 | 4 | 209,04 | 47,27 |
| III/II | 297 | l | n | 4-8 | 4 | 192,53 | 48,95 |
| III/II | 323 | r | n | 4-8 | 4 | 213,6 | 49,92 |
| III/II | 144 | l | y | 8-14 | 5 | 223,64 | 48,5 |
| III/II | 294 | r | y | 8-14 | 5 |  | 57,18 |
| III | 226 | r | y | 2-3 | 2 |  | 47,09 |
| III | 328b | l | y | 2-3 | 2 |  | 48,95 |
| III | 151 | r | n | 3-4 | 3 | 202,91 | 46,9 |
| III | 152 | l | y | 3-4 | 3 |  | 48,43 |
| III | 194 | l | y | 3-4 | 3 | 222,2 | 53,1 |
| III | 214 | l | n | 3-4 | 3 | 198,71 | 51,77 |
| III | 274 | l | y | 3-4 | 3 |  | 46,34 |
| III | 279 | r | n | 3-4 | 3 | 220,31 | 51,65 |
| III | 423 | r | n | 3-4 | 3 | 196,37 | 52,31 |
| III | 460 | l | y | 3-4 | 3 |  | 43,92 |
| III | 461 | r | n | 3-4 | 3 | 203,04 | 49,6 |
| III | 121 | r | y | 4-8 | 4 | 196 | 52,25 |
| III | 143 | l | y | 4-8 | 4 |  | 55,44 |
| III | 146 | r | n | 4-8 | 4 | 201,32 | 44,59 |
| III | 189 | l | n | 4-8 | 4 | 201,71 | 52,18 |
| III | 215 | r | n | 4-8 | 4 | 218,82 | 53,72 |
| III | 225 | l | n | 4-8 | 4 | 206,02 | 47,56 |
| III | 253 | r | n | 4-8 | 4 | 190,25 | 49,16 |
| III | 274 | r | n | 4-8 | 4 | 200 | 45,29 |
| III | 370 | l | y | 4-8 | 4 | 227,56 | 49,99 |
| III | 397 | r | n | 4-8 | 4 | 200,25 | 44,41 |
| III | 416 | r | y | 4-8 | 4 | 199,49 | 42,56 |
| III | 444 | r | y | 4-8 | 4 | 202,17 | 46,11 |
| III | 174 | r | y | 8-14 | 5 | 200,26 | 46,85 |
| III | 217 | r | y | 8-14 | 5 |  | 58,3 |
| III | 217 | r | n | 8-14 | 5 | 197,46 | 54,76 |
| III | 250 | r | y | 8-14 | 5 | 201,66 | 48,29 |
| III | 342 | l | n | 8-14 | 5 | 195,96 | 43,9 |
| III | 414 | r | y | 8-14 | 5 |  | 57,17 |
| III | 81 | r | y | 15 | 6 | 198,3 | 54,01 |
| III | 104 | r | y | 15 | 6 | 212,33 | 56,22 |
| III | 192 | l | y | 15 | 6 | 217,18 | 57,67 |
| III | 199 | r | y | 15 | 6 | 211,45 | 44,91 |
| III | 263 | r | y | 15 | 6 | 209,07 | 63,97 |
| III | 317 | l | y | 15 | 6 | 197,16 | 55,05 |

*Visible line of fusion
