# Supplementary material for: Ageing Cattle: The Use of Radiographic Examinations on Cattle Metapodials from Eketorp Ringfort on the Island of Öland in Sweden
Source: PLoS One. 2015 Sep 3;10(9):e0137109. doi: 10.1371/journal.pone.0137109 (PMC4559407; doi:10.1371/journal.pone.0137109)
Supplement: S3 Table — (DOCX) [file pone.0137109.s003.docx]

S3 Table: Summarized data based on Boessneck et al. (1979:66, diagram 7,

in Eketorp. Befestigung und Siedlung auf Öland/Schweden. Die Fauna.

Lieber Tryck Stockholm,pp 504) measurements on bone elements from

Eketorp ringfort phase II and III.

|  | Phase | Cow | Oxen/bull |
| --- | --- | --- | --- |
|  |  |  |  |
| Pelvis | II | 98 | 28 |
|  | III | 243 | 78 |
| **Total** | **447** | **341** | **106** |
|  |  |  |  |
| Metacarpals | II | 71 | 18 |
|  | III | 209 | 70 |
| **Total** | **368** | **280** | **88** |
|  |  |  |  |
| Metatarsals | II | 74 | 24 |
|  | III | 177 | 60 |
| **Total** | **335** | **251** | **84** |
|  |  |  |  |
